# Comparative efficacy of zero-profile implant and conventional cage-plate implant in the treatment of single-level degenerative cervical spondylosis: a systematic review and meta-analysis

**DOI:** 10.1186/s13018-024-04729-5

**Published:** 2024-06-19

**Authors:** Peng Zhang, Hongyu Zheng, Jun Luo, Jie Xu

**Affiliations:** 1 Shengli Clinical Medical College of Fujian Medical University, Fuzhou, 350000 China; 2 Department of Anesthesiology, Xiangyang Central Hospital, Affiliated Hospital of Hubei University of Arts and Science, Xiangyang, 441021 Hubei China; 3 Department of Orthopedics, Fujian Provincial Hospital, Fujian Medical University, Fuzhou, 350000 China

**Keywords:** Anterior cervical decompression and fusion, Zero-profile, Cage plate, Single level, Meta-analysis

## Abstract

**Background:**

In recent years, the zero-profile implant (Zero-p) has emerged as a promising internal fixation technique. Although studies have indicated its potential superiority over conventional cage-plate implant (Cage-plate) in the treatment of degenerative cervical spondylosis, there remains a lack of definitive comparative reports regarding its indications, safety, and efficacy.

**Methods:**

A computerized search was conducted on English and Chinese databases, including PubMed, Web of Science, Cochrane Library, EMBASE, CNKI, Wanfang and VIP. Additionally, a manual search was meticulously carried out on Chinese medical journals, spanning from the inception of the respective databases until August 2023. The meta-analysis utilized a case–control study approach and was executed through the utilization of RevMan 5.3 software. Stringent quality evaluation and data extraction procedures were implemented to guarantee the reliability and validity of the findings.

**Results:**

Nine high-quality studies with 808 patients were included. Meta-analysis showed that the operation time (MD = − 13.28; 95% CI (− 17.53, − 9.04), *P* < 0.00001), intraoperative blood loss (MD = − 6.61; 95% CI (− 10.47, − 2.75), *P* = 0.0008), incidence of postoperative dysphagia at various time points: within the first month after surgery (OR = 0.36; 95% CI (0.22, 0.58), *P* < 0.0001), 1–3 months after surgery (OR = 0.20; 95% CI (0.08, 0.49), *P* = 0.0004), the final follow-up (OR = 0.21; 95% CI (0.05, 0.83), *P* = 0.003) and the rate of postoperative adjacent disc degeneration (OR = 0.46; 95% CI (0.25, 0.84), *P* = 0.01) were significantly lower in the Zero-p group than in the Cage-plate group. Additionally, was also significantly lower in the Zero-p group. However, there were no significant differences in the JOA score, the final follow-up NDI score, surgical segmental fusion rate, postoperative height of adjacent vertebrae, or postoperative subsidence rate between the two groups.

**Conclusion:**

In summary, when treating single-segment degenerative cervical spondylosis, both internal fixation techniques are reliable and effective. However, Zero-P  implant offer several advantages over cage-plate implant, including shorter operation duration, less intraoperative blood loss, reduced postoperative dysphagia, and slower adjacent disc degeneration. Additionally, Zero-P implant has a broader application space, making them a preferred choice in certain cases.

## Background

In recent years, zero-profile implants (Zero-p) has emerged as an internal fixation technique that has demonstrated promising results in the treatment of degenerative cervical spondylosis. According to studies [1, 2], Zero-p exhibits numerous advantages over cage-plate implants (Cage-plate) techniques. Furthermore, it has been shown to effectively minimize postoperative dysphagia and mitigate the risk of adjacent segment degeneration [3]. Given its superior therapeutic outcomes in treating degenerative cervical spondylosis, Zero-p has gradually gained acceptance and application in surgical procedures within this domain.

With the ongoing advancements in minimally invasive surgical techniques for cervical spondylosis, anterior cervical decompression and fusion (ACDF) has emerged as an effective method for decompressing the spinal cord and nerve roots, while also facilitating cervical fusion, thereby enhancing the stability of the cervical spine structure [4]. Despite the progress made, there remains a scarcity of comparative studies comparing Zero-p ACDF and Cage-plate ACDF surgeries. Furthermore, there is a dire need for comprehensive and unified reports on the long-term efficacy and potential complications associated with these procedures. Existing studies have demonstrated promising clinical outcomes with the use of Zero-p ACDF [5, 6], yet clear comparative data on indications, safety, and efficacy are lacking. To address this gap in knowledge and provide further clinical evidence, this systematic review and meta-analysis aims to analyze and compare the clinical outcomes and postoperative complications associated with Zero-p and Cage-plate techniques in the surgical management of single-segment degenerative cervical spondylosis. Our aim is to furnish clinicians with robust data support to facilitate informed decisions regarding the implementation of these two internal fixation surgeries in clinical practice.

## Methods

### Search strategy

The screening process adheres strictly to the PRISMA guidelines for conducting systematic reviews, as outlined in reference [7]. A comprehensive search was conducted using computer-assisted methods on various databases, including English and Chinese repositories such as PubMed, Web of Science, Cochrane Library, EMBASE, CNKI, Wanfang, and VIP databases. To ensure a thorough search, relevant literature published in Chinese medical journals was manually reviewed. This retrieval encompassed the entire duration from the establishment of these databases up to August 2023.

Sophisticated retrieval strategies were employed, utilizing subject terms and keywords such as "Zero-p," "Zero-profile," "ROI-C," "Cage-plate," "Standalone anchored spacer," "anchored Cage," "anchored fusion," "no-profile," and "ACDF." To ensure the inclusion of as many randomized controlled studies as possible, the references cited in the searched literature were also examined, thereby enhancing the comprehensiveness of the data. Furthermore, studies originating from the same institutions were carefully evaluated to prevent any duplication in data collection.

### Inclusion criteria

The studies encompassed in this analysis were clinical investigations pertaining to the surgical treatment of cervical spondylosis, specifically evaluating the use of Zero-p or Cage-plate techniques during anterior vertebral decompression and fusion procedures. These studies adhered to six predefined criteria: (1) surgical interventions were restricted to decompression of a single intervertebral space and fusion of the adjacent vertebral bodies above and below; (2) a minimum follow-up duration of 18 months was required; (3) studies including  patients with a history of neck trauma, neurological, or spinal cord injuries, as well as any other systemic disorders, were excluded; (4) the Newcastle–Ottawa Scale (NOS) [8] was employed to assess the quality of cohort studies, with a minimum score of 4 required for inclusion; (5) the sample size had to be more than 40 subjects overall or include at least 20 subjects in each comparison group; (6) only Chinese-language articles published in high-quality journals indexed by the Chinese Science Citation Database (CSCD) were considered for inclusion in the present analysis.

### Literature selection and quality evaluation

A thorough search was conducted by two investigators, ensuring that the retrieved information adhered strictly to the set inclusion and exclusion criteria. All potential sources of literature that met the inclusion criteria underwent comprehensive textual analysis. To determine the eligibility of RCTs for inclusion in the study, a rigorous quality evaluation was conducted, referencing the recommended criteria outlined by the Cochrane system. For the cohort study that encompassed observational studies, the Newcastle–Ottawa Scale (NOS) was employed to meticulously assess the quality of the studies across three key dimensions: selection, comparability, and results. A final cross-check was performed to ensure the accuracy and consistency of the findings.

### Statistical analysis

The Review Manager 5.3 software was utilized for the purpose of analysis. Measurement of data, including operation time, intraoperative blood loss, Japanese Orthopaedic Association (JOA) score, Neck Disability Index (NDI) score, and postoperative height of adjacent vertebrae, was conducted using weighted mean differences (MD) and 95% confidence intervals (CI). Dichotomous variables, such as postoperative subsidence rate, the incidence of dysphagia, incidence of postoperative adjacent segment ossification, and incidence of adjacent segment degeneration, were represented as odds ratios (OR) along with their respective 95% CI. Heterogeneity among studies was assessed using the *I*^2^ statistic. When the results exhibited low heterogeneity (*P* > 0.1, *I*^2^ ≤ 50%), a fixed-effects model was utilized. Conversely, in the presence of high heterogeneity among studies (*P* < 0.1, *I*^2^ > 50%), the random-effects model was applied to mitigate clinical heterogeneity. *P* < 0.05 was considered statistically significant.

## Results

### Search results

**Fig. 1:**
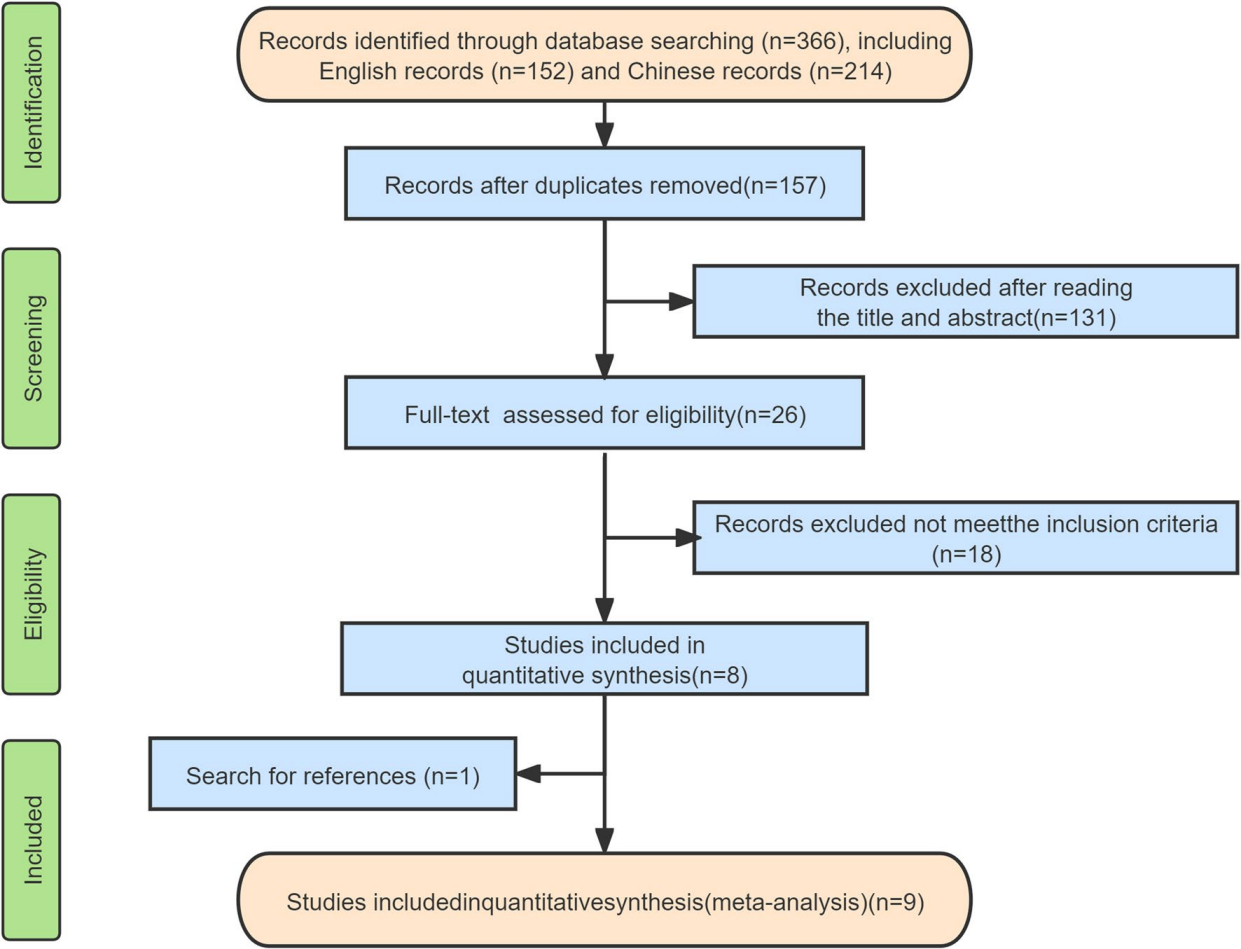
Flow diagram of study selection

After a thorough screening process, we selected seven English studies [9, 10, 13–17] and two Chinese studies [11, 12] for inclusion in our study. These articles included a total of 808 patients with single-level degenerative cervical spondylosis, with 353 patients assigned to the Zero-p group and 455 patients assigned to the Cage-plate group. The specific screening process is outlined in Fig. 1, while Table 1 provides an overview of the basic characteristics of the included studies. Additionally, one randomized controlled trial (RCT) was included in our analysis, and its quality evaluation yielded a score of 3 points, indicating high quality. Table 2 presents the quality evaluation of the eight retrospective cohort studies included in our meta-analysis.

**Table 1:** Characteristics of the included studies

Study	Published year	Study type	N (male/female, case)		Mean age (years)		Follow-up period (months)	
			Zero­p	Cage-plate	Zero­p	Cage-plate	Zero­p	Cage-plate
Zhang XB [9]	2021	Retrospective cohort	40/34	68/48	50.14 ± 6.05	50.29 ± 9.06	34.07 ± 3.20	36.50 ± 6.28
He SJ [10]	2021	Retrospective cohort	19/23	20/25	62.59 ± 8.21	61.15 ± 7.52	26.6 ± 3.3	27.1 ± 3.5
Yang JS [11]	2020	Retrospective cohort	26/24	29/21	58.3	58.6	Minimum 24	
Wang F [12]	2019	Retrospective cohort	13/8	14/7	49.19 ± 7.26	50.27 ± 8.75	18	
Noh SH [13]	2018	Retrospective cohort	11/25	31/40	55.64 ± 10.31	55.06 ± 11.13	32.7 ± 17.5	
Shao HY [14]	2016	Retrospective cohort	38/25	45/31	47.6 ± 6.4	50.3 ± 8.2	23.6 ± 4.5	25.2 ± 4.8
Cho HJ [15]	2015	Retrospective cohort	12/9	19/10	56.1 ± 12.0	55.2 ± 10.4	Minimum 24	
Nemoto O [16]	2014	RCT	21/3	21/1	40.9 ± 7.2	41.6 ± 7.0	Minimum 24	
Wang ZD [17]	2014	Retrospective cohort	11/11	10/15	50.86 ± 8.79	53.68 ± 8.96	33.59 ± 5.52	33.16 ± 5.97

**Table 2:** Methodological quality-based evaluation of the 8 included retrospective cohort studies

Study included	Selection	Comparability	Exposure/outcome	Quality scores
Zhang XB [9]	3	2	3	8
He SJ [10]	3	2	3	8
Yang JS [11]	3	2	3	8
Wang F [12]	3	2	3	8
Noh SH [13]	3	2	3	8
Shao HY [14]	3	2	3	8
Cho HJ [15]	3	2	3	8
Wang ZD [17]	3	2	3	8

## Outcomes

### Intraoperative findings

#### Operation time

**Fig. 2:**
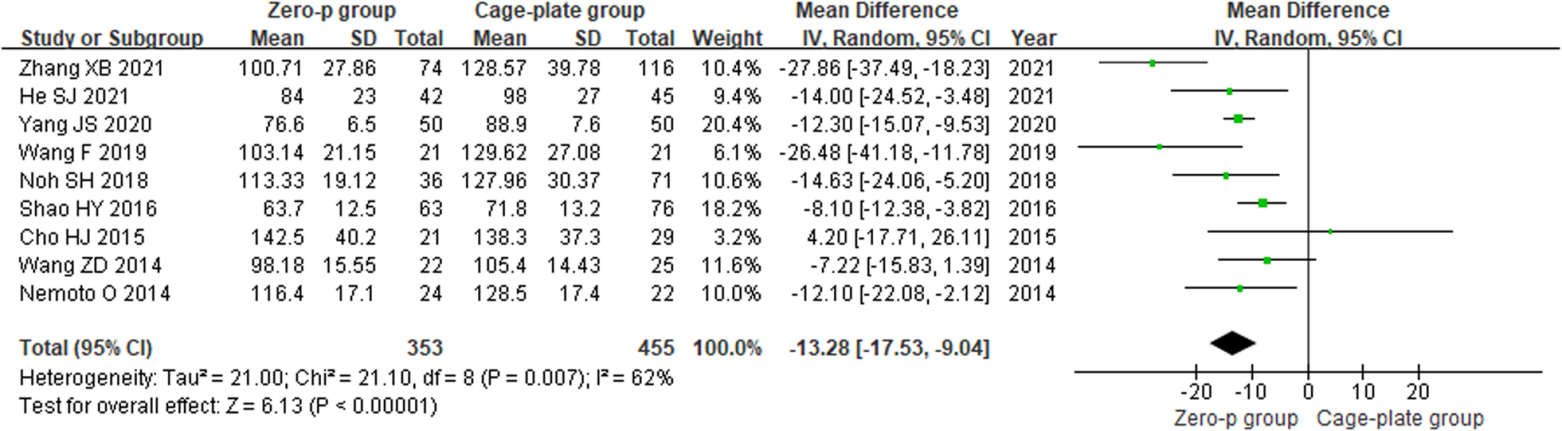
Forest plot of operation time (SD, standard deviation; IV, inverse-variance method; CI, confidence interval; df, degree of freedom)

Nine studies [9–17] reported the operation time. There was a significant heterogeneity in the literature (*P* = 0.007, *I*^2^ = 62%). Meta-analysis was performed using random-effect model, and the result indicated that the Zero-p group had a significantly shorter operation time compared to the Cage-plate group (MD = − 13.28; 95% CI (− 17.53, − 9.04), *P* < 0.00001). The corresponding forest plot was shown in Fig. 2.

### Intraoperative blood loss

**Fig. 3:**
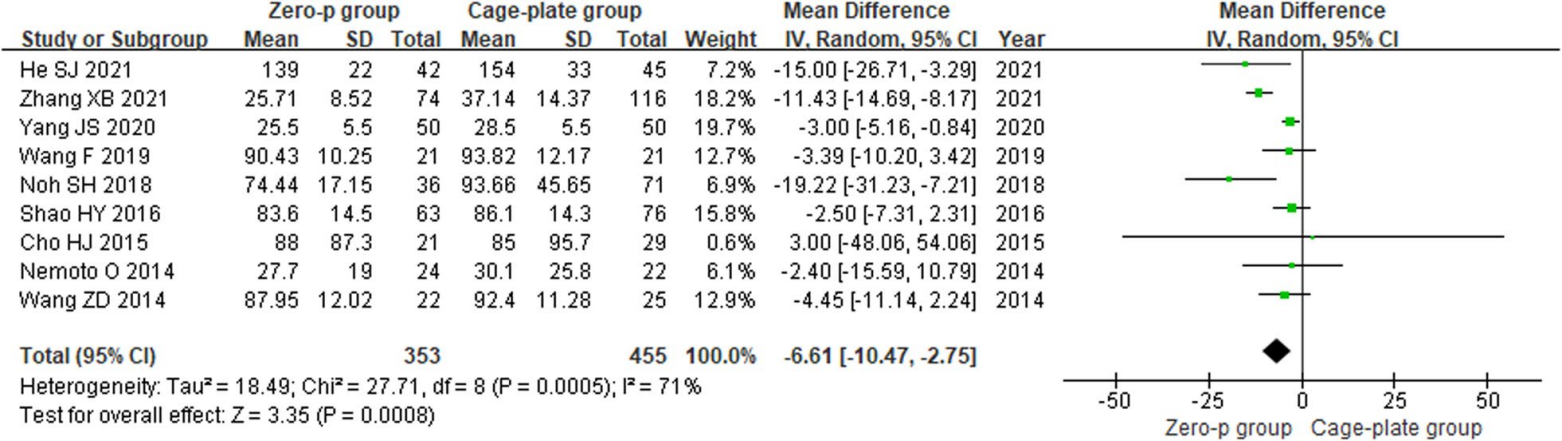
Forest plot of intraoperative blood loss

Nine studies [9–17] reported the intraoperative blood loss. There was a significant heterogeneity in the literature (*P* = 0.0005, *I*^2^ = 71%). Meta-analysis was performed using random-effect model, and the result indicated that the Zero-p group had a significantly less intraoperative blood loss compared to the Cage-plate group (MD = − 6.61; 95% CI (− 10.47, − 2.75), *P* = 0.0008). The corresponding forest plot was shown in Fig. 3.

### Clinical effects

#### Postoperative JOA score

**Fig. 4:**
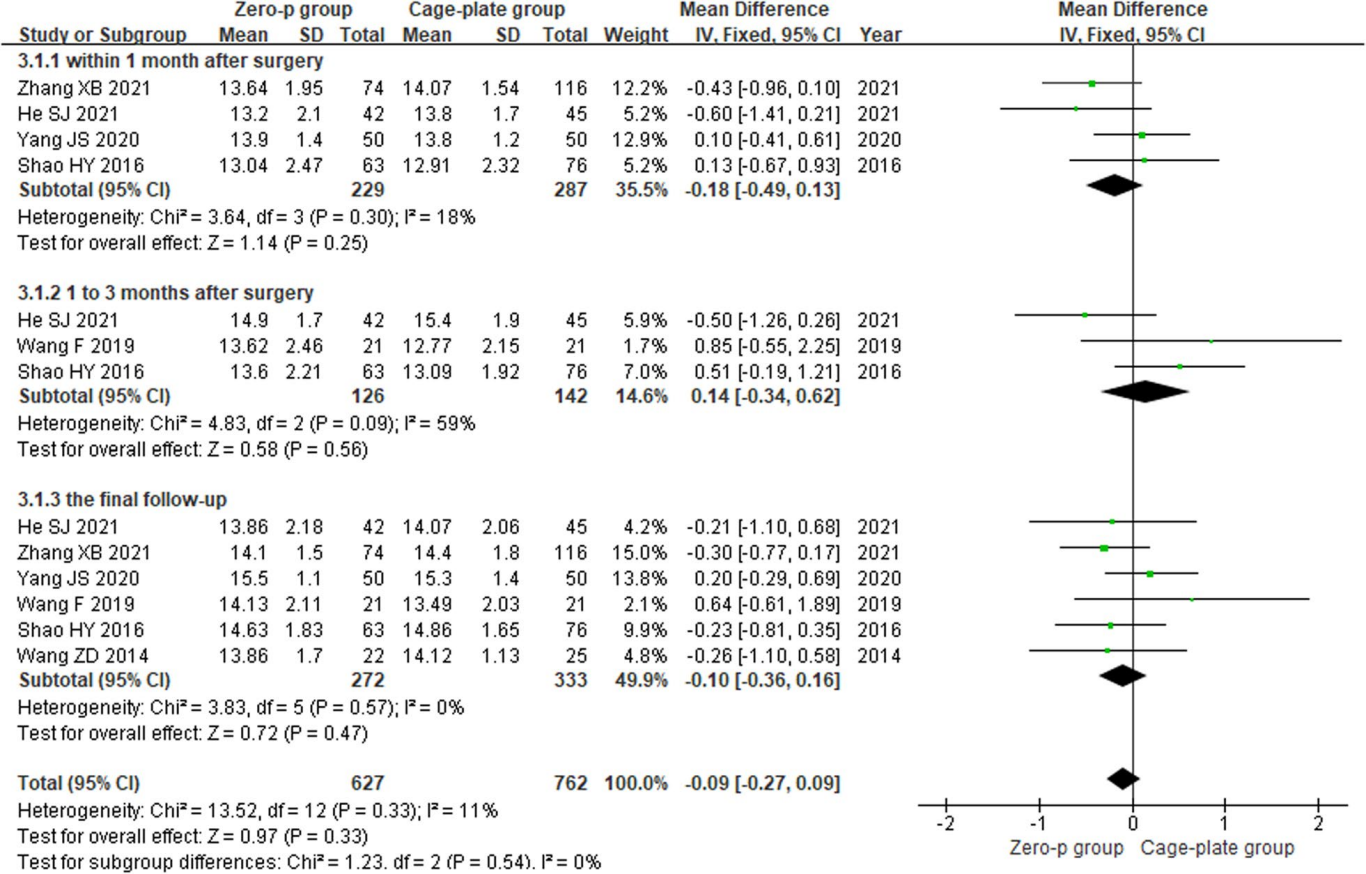
Forest plot of the postoperative JOA score

Four studies [9–11, 14] reported the JOA score within 1 month after surgery, three studies [10, 12, 14] reported at 1–3 months after surgery, and six articles [9–12, 14, 17] reported at the final follow-up. There was low heterogeneity in the literature (*P* = 0.33, *I*^2^ = 11%). Meta-analysis was performed using fixed-effect model, and the results of subgroup analysis showed that there was no significant difference in JOA score between the Zero-p and Cage-plate group within 1 month after surgery (MD = − 0.18; 95% CI (− 0.49, 0.13), *P* = 0.25), 1–3 months after surgery (MD = − 0.14; 95% CI (− 0.34, 0.62), *P* = 0.56) and the final follow-up (MD = − 0.10; 95% CI (− 0.36, 0.16), *P* = 0.47). The corresponding forest plot was shown in Fig. 4.

#### Postoperative NDI score

**Fig. 5:**
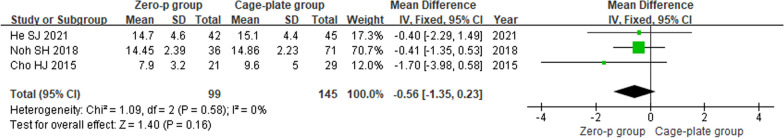
Forest plot of postoperative NDI scores

Three studies [10, 13, 15] reported the NDI score at the final follow-up. There was no heterogeneity in the literature (*P* = 0.58, *I*^2^ = 0%). Meta-analysis was performed using fixed-effect model, and the results of subgroup analysis showed that there was no significant difference in NDI score between the Zero-p and Cage-plate group at the final follow-up (MD = − 0.56; 95% CI (− 1.35, 0.23),* P* = 0.16). The corresponding forest plot was shown in Fig. 5.

### Imaging evaluation

#### Surgical segmental fusion rate

**Fig. 6:**
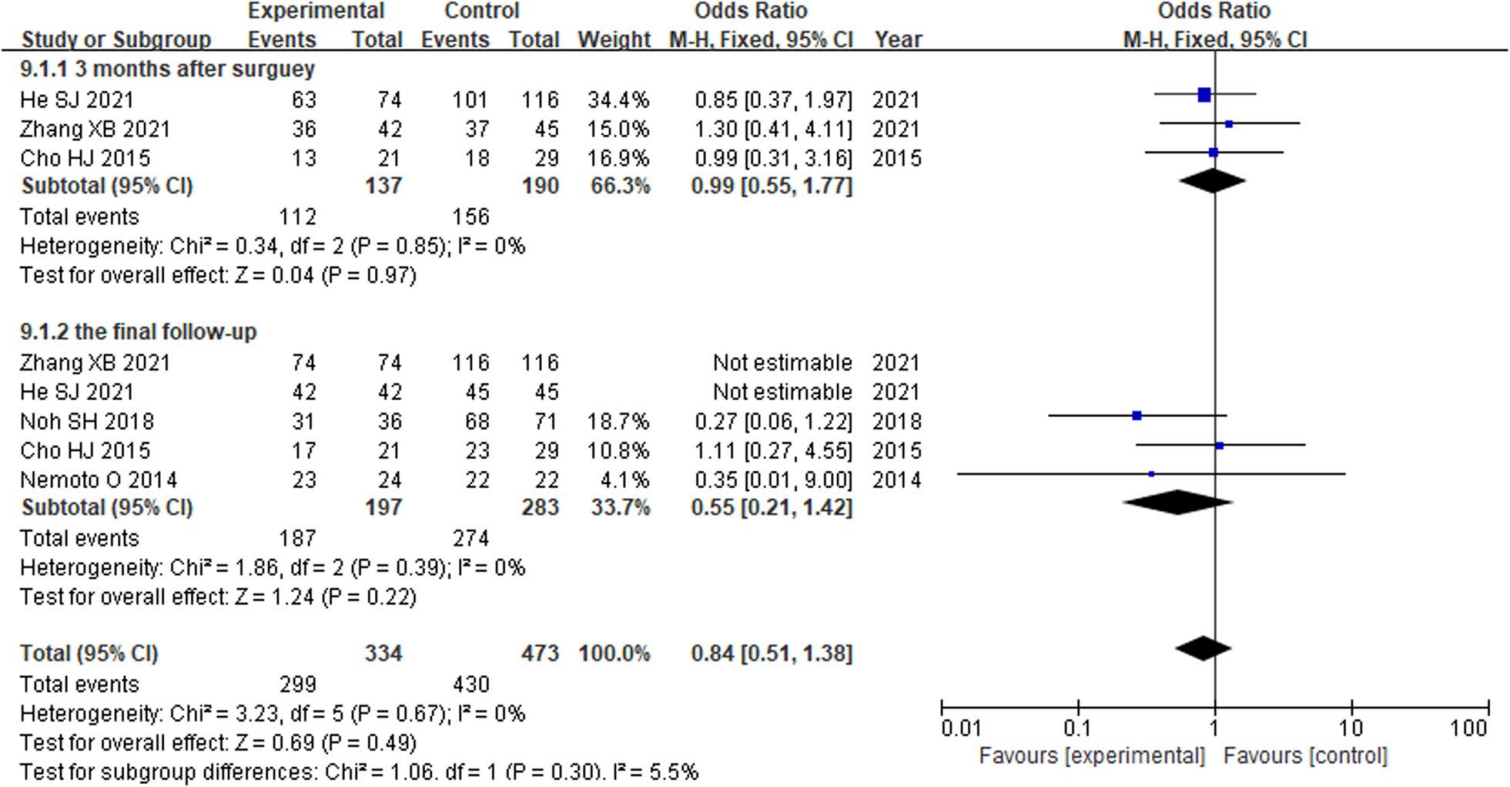
Forest plot of surgical segmental fusion rate

Three studies [9, 10, 15] reported the surgical segment fusion rate at 3 month after surgery. Five articles [9, 10, 13, 15, 16] reported the surgical segment fusion rate at the final follow-up. There was no heterogeneity in the literature (*P* = 0.67, *I*^2^ = 0%). Meta-analysis was performed using fixed-effect model, and the results of subgroup analysis showed that there was no significant difference in the surgical segment fusion rate between the two groups at 3 months after operation (OR = 0.99; 95% CI (0.55, 1.77),* P* = 0.97). And at the final follow-up (OR = 0.55; 95% CI (0.21, 1.42),* P* = 0.22). The corresponding forest plot was shown in Fig. 6.

#### Postoperative height of adjacent vertebrae

**Fig. 7:**
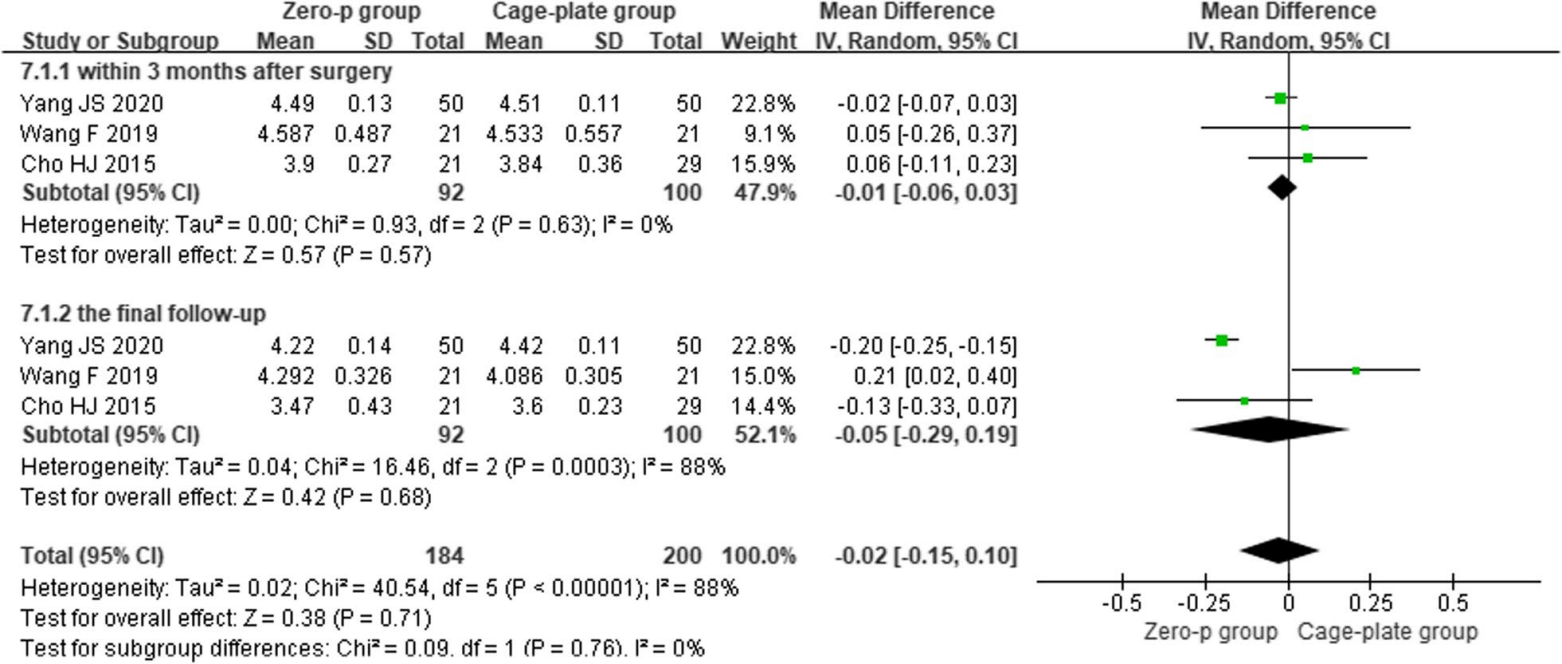
Forest plot of postoperative height of adjacent vertebrae

Three studies [11, 12, 15] reported the NDI score within 3 month after surgery and the final follow-up. There was high heterogeneity in the literature (*P* < 0.00001, *I*^*2*^ = 88%). Meta-analysis was performed using random-effect model, and the results of subgroup analysis showed that there was no significant difference in NDI score between the Zero-p and Cage-plate group within 3 month after surgery(MD = − 0.01, 95% CI (− 0.06, 0.03), *P* = 0.63), and the final follow-up (MD = − 0.05, 95% CI (− 0.29, 0.19), *P* = 0.68). The corresponding forest plot was shown in Fig. 7.

#### Postoperative subsidence rate

**Fig. 8:**
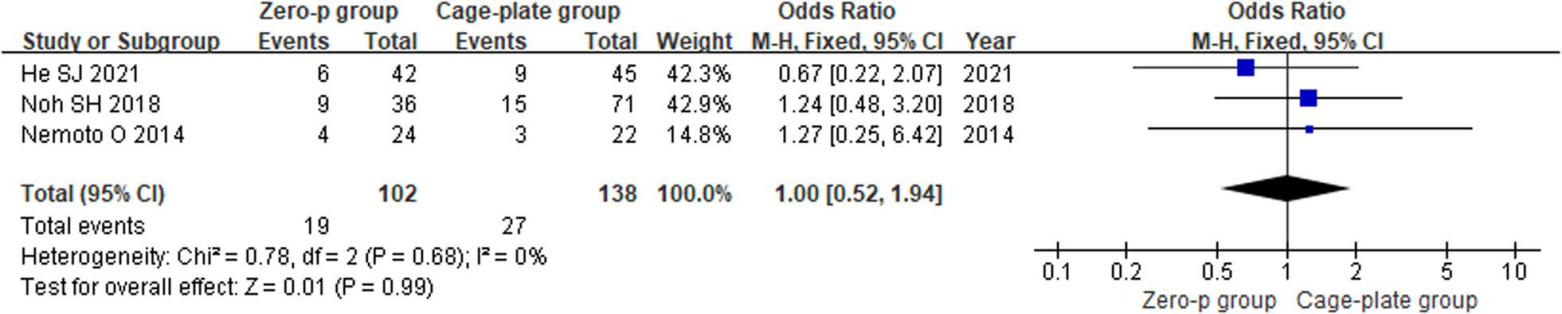
Forest plot of postoperative settlement rate

Three studies [10, 13, 16] reported the postoperative subsidence rate.There was no heterogeneity in the literature (*P* = 0.68, *I*^2^ = 0%). Meta-analysis was performed using fixed-effect model, and the results of subgroup analysis showed that there was no significant difference in postoperative subsidence rate between the Zero-p and Cage-plate group (OR = 1.00; 95% CI (0.52, 1.94),* P* = 0.99). The corresponding forest plot was shown in Fig. 8.

**Fig. 9:**
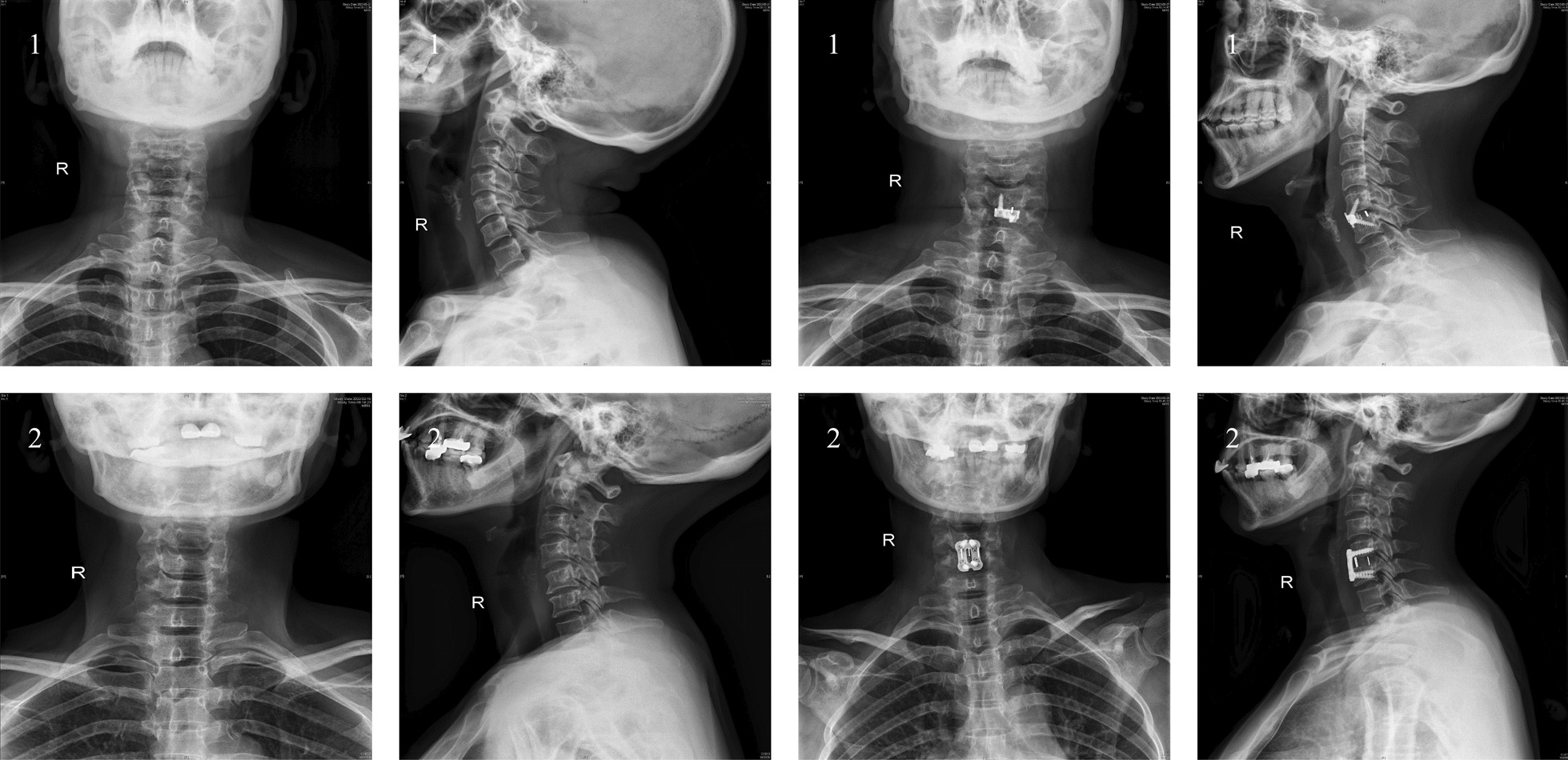
Postoperativel coronal and sagittal cervical X-ray with Zero-P or Cage-plate surgery. **1A**, **1B** Postoperative cervical X-ray with Zero-p surgery. **1C**, **1D** X-ray at postoperative 1 month with Zero-p surgery. **2A**, **2B** Postoperative cervical X-ray with Cage-plate surgery. **2C**, **2D** X-ray at postoperative 1 month with Cage-plate surgery

A comparation of radiographs depicting the utilization of zero-profile implants versus conventional cage-plate implants for the treatment of single-level degenerative cervical spondylosis is presented in Fig. 9.

### Postoperative complications

#### Incidence of postoperative dysphagia

**Fig. 10:**
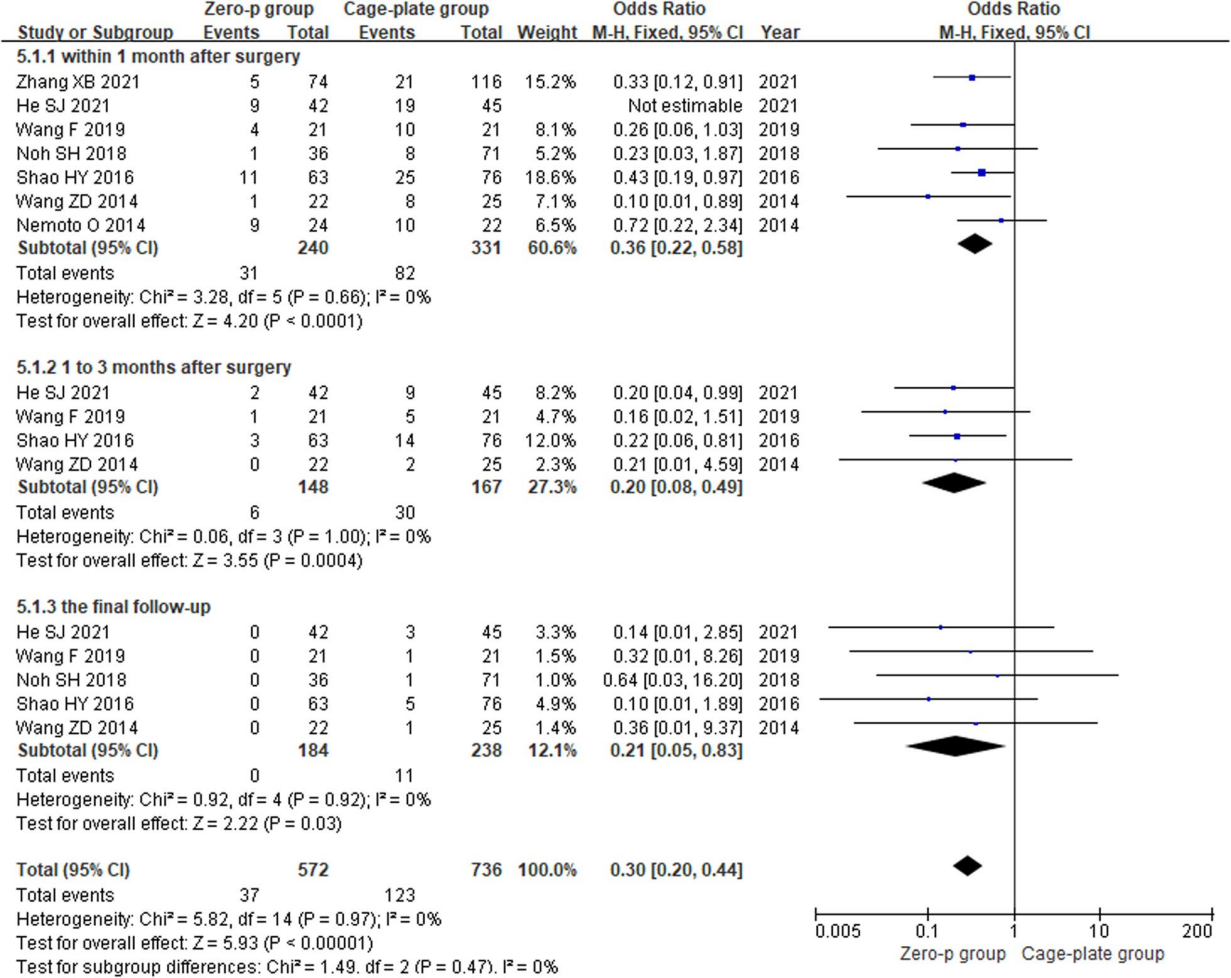
Comparison of incidence of dysphagia

Seven studies [9, 10, 12–14, 16, 17] reported the incidence of postoperative dysphagia within 1 month after surgery. Four articles [10, 12, 14, 17] reported the incidence of postoperative dysphagia within 1–3 months after surgery, and five articles [10–14, 17] reported the incidence of postoperative dysphagia at the final follow-up. There was low heterogeneity in the literature (*P* = 0.97, *I*^2^ = 0%). Meta-analysis was performed using fixed-effect model, and the results of subgroup analysis showed that there was no significant difference in the incidence of postoperative dysphagia between the Zero-p and Cage-plate group within 1 month after surgery (OR = 0.36; 95% CI (0.22, 0.58),* P* < 0.0001), 1–3 months after surgery (OR = 0.20; 95% CI (0.08, 0.49),* P* = 0.0004) and the final follow-up (OR = 0.21; 95% CI (0.05, 0.83),* P* = 0.003). The corresponding forest plot was shown in Fig. 10.

### Postoperative rate of adjacent disc degeneration

**Fig. 11:**
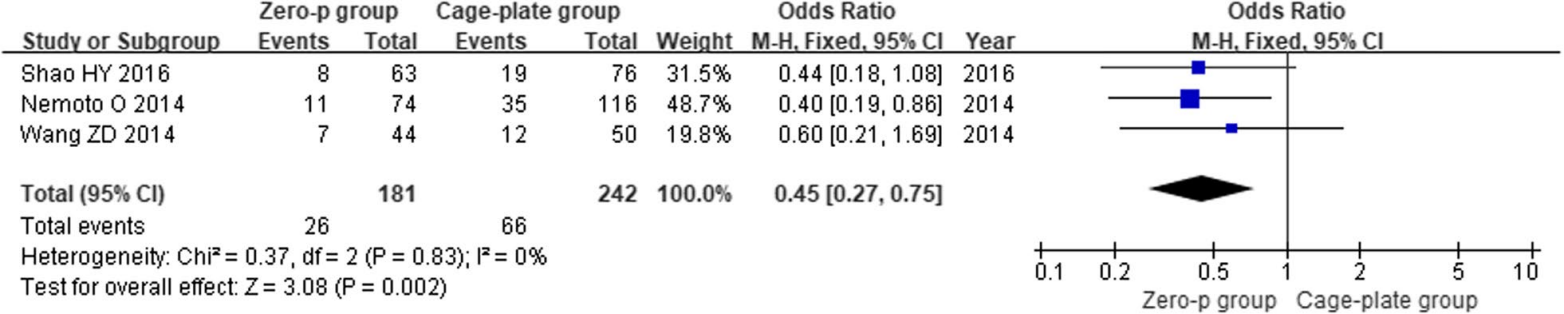
Comparison of the postoperative adjacent disc degeneration rate

Three studies [14, 16, 17] reported the postoperative adjacent disc degeneration rate. There was low heterogeneity in the literature (*P* = 0.83, *I*^2^ = 0%). Meta-analysis was performed using fixed-effect model, and the results analysis showed that there was significant difference in the postoperative adjacent disc degeneration rate between the Zero-p and Cage-plate group (OR = 0.45; 95% CI (0.27, 0.75),* P* = 0.002). The corresponding forest plot was shown in Fig. 11.

## Discussion

In recent years, due to the widespread use of electronic devices and an increase in desk-based work, cervical spondylosis has become increasingly prevalent in clinical settings [18]. Furthermore, the proportion of patients requiring surgical intervention to alleviate their symptoms has also been on the rise. Consequently, the selection of the surgical plan is crucial in maintaining the future quality of life for these patients [4]. Among the surgical options, ACDF has gradually emerged as the most frequently utilized anterior approach for the treatment of degenerative cervical spondylosis due to its minimal invasiveness [19, 20].

A comprehensive study encompassing nine articles was conducted, encompassing a total of 353 patients in the Zero-p group and 455 patients in the Cage-plate group. The findings revealed that the operation time, intraoperative blood loss, incidence of postoperative dysphagia, rate of postoperative adjacent disc degeneration were significantly reduced in the Zero-p group compared to the Cage-plate group. However, the meta-analysis did not yield any significant differences in the JOA score, the final follow-up NDI score, Surgical segmental fusion rate, postoperative height of adjacent vertebrae or postoperative subsidence rate between the two groups.

Cage-plate is a well-regarded surgical procedure in the context of ACDF. When compared to traditional open fusion techniques, Cage-plate offers several advantages, including reduced trauma, accelerated recovery and minimal impact on spinal stability. This fusion method not only provides structural support but also facilitates bone healing. Additionally, the titanium plate screw internal fixation system serves to stabilize the surgical site, maintaining the integrity of the procedure. The fusion and fixation of the upper and lower vertebral bodies within the affected intervertebral space serve to prevent the displacement or migration of the fusion cage. However, this stabilization comes with a cost: a loss of local range of motion (ROM). Consequently, the ROM and intervertebral pressure of the adjacent segments are forced to increase, leading to a higher risk of adjacent segment degeneration [21]. Intraoperative manipulation and stripping of soft tissues can result in increased intraoperative blood loss, which can obscure the surgical field and contribute to postoperative soft tissue edema, hoarseness and dysphagia among other complications [22–24]. Some studies suggest that the thickness of the plate may be a contributing factor to prevertebral soft tissue thickening, dysphagia and hoarseness [15, 25, 26]. Especially when the distance between the edge of plate and adjacent segment is less than 5 mm, the incidence of adjacent disc degeneration will increase [27]. Furthermore, the titanium plate's contact with the adjacent intervertebral space can lead to ossification and degeneration, particularly when the plate is positioned close to the adjacent disc. This can manifest as a range of clinical symptoms, including labial hyperplasia in the affected area [28]. To address these challenges, the Zero-p fusion cage has been developed. Its innovative design and structure aim to minimize the thickness of soft tissue anterior to the vertebral body, thereby reducing the incidence of dysphagia. Additionally, the Zero-P cage fulfills the functions of fixation, support and fusion, effectively compensating for the limitations of traditional Cage-plate techniques [29].

Patients with severe osteoporosis should avoid using a Zero-p fusion device. When the curved insert is positioned at a specific, consistent angle within the vertebral bodies, it facilitates stress distribution and decreases sedimentation rates. However, this approach carries the risk of internal fixator loosening and displacement, particularly prevalent among osteoporosis patients. Additionally, meticulous attention to endplate management during the surgical procedure is crucial to prevent fusion cage settlement and enhance local stability. Furthermore, Zero-p fusion surgery is contraindicated for patients with cervical spondylosis complicated by congenital cervical canal stenosis, ossification of the posterior longitudinal ligament (OPLL) or multiple significant compressions of the ventral and dorsal cervical medulla, as referenced in studies [30, 31].

## Conclusions

In summation, Zero-p emerges as a reliable and effective surgical approach for managing degenerative cervical spondylosis, when compared to Cage-plates. Both methods exhibit the benefits of minimized trauma, accelerated recovery and impressive therapeutic outcomes. Nevertheless, the utilization of cage plates is associated with a higher occurrence of dysphagia and adjacent disc degeneration. In contrast, Zero-­p significantly minimizes these complications, offers a shorter surgical duration, minimizes intraoperative blood loss and demonstrates superior long-term NDI scores. Therefore, it is advisable for clinicians to consider Zero-p as a preferred treatment option for degenerative cervical spondylosis, subject to suitable conditions.

## Data Availability

The patient data adopted are from the internet.

## Abbreviations

Zero-p: Zero-profile
Cage-plate: Conventional cage-plate implant
ACDF: Anterior cervical decompression and bone graft fusion
CSCD: High-quality Chinese Science Citation Database
NOS: Newcastle‒Ottawa Scale
CI: Confidence intervals
JOA: Japanese Orthopaedic Association
NDI: Neck Disability Index
OR: Odds ratio
SD: Standard deviation
IV: Inverse-variance method
CI: Confidence interval
df: Degree of freedom
RCT: Randomized controlled trial
ROM: Range of motion
OPLL: Ossificine of posterior longtitudining ligareent
